# Small extracellular vesicles: from promoting pre-metastatic niche formation to therapeutic strategies in breast cancer

**DOI:** 10.1186/s12964-022-00945-w

**Published:** 2022-09-12

**Authors:** Xiaoxiao Chen, Jiamei Feng, Weili Chen, Shijun Shao, Li Chen, Hua Wan

**Affiliations:** 1 Department of Breast, Shuguang Hospital, Shanghai University of Traditional Chinese Medicine, Shanghai, 200001 China; 2 Department of Breast, Yueyang Hospital Integated Traditional Chinese and Western Medicine, Shanghai University of Traditional Chinese Medicine, Shanghai, 200080 China; 3 Laboratory of Immunopharmacology, State Key Laboratory of Drug Research, Shanghai Institute of Materia Medica, Chinese Academy of Sciences, Shanghai, 201203 China; 4 University of Chinese Academy of Sciences, Beijing, 100049 China

**Keywords:** Breast cancer, sEVs, Pre-metastatic niche, Metastasis, MiRNA, Immunotherapy

## Abstract

Breast cancer is the most common cancer in females, and to date, the mortality rate of breast cancer metastasis cannot be ignored. The metastasis of breast cancer is a complex, staged process, and the pattern of metastatic spread is not random. The pre-metastatic niche, as an organ-specific home for metastasis, is a favourable environment for tumour cell colonization. As detection techniques improve, the role of the pre-metastatic niche in breast cancer metastasis is being uncovered. sEVs (small extracellular vesicles) can deliver cargo, which is vital for the formation of pre-metastatic niches. sEVs participate in multiple aspects of creating a distant microenvironment to promote tumour invasion, including the secretion of inflammatory molecules, immunosuppression, angiogenesis and enhancement of vascular permeability, as well as regulation of the stromal environment. Here, we discuss the multifaceted mechanisms through which breast cancer-derived sEVs contribute to pre-metastatic niches. In addition, sEVs as biomarkers and antimetastatic therapies are also discussed, particularly their use in transporting exosomal microRNAs. The study of sEVs may provide insight into immunotherapy and targeted therapies for breast cancer, and we also provide an overview of their potential role in antitumour metastasis.

**Video Abstract**

**Supplementary Information:**

The online version contains supplementary material available at 10.1186/s12964-022-00945-w.

## Background

As of 2020, breast cancer has surpassed lung cancer as the most common type of cancer among women worldwide [1], accounting for approximately 30% of female cancers with a mortality rate of 15% [2]. Metastasis remains the biggest cause of death for breast cancer patients (approximately 90%) [3]. The 5-year survival rate of metastatic breast cancer is significantly reduced compared with that of nonmetastatic breast cancer, and the median overall survival of metastatic triple-negative breast cancer (TNBC) is only 1 year [4, 5]. Surgery and adjuvant therapy can cure well-confined primary tumours, but metastatic disease is largely incurable due to drug resistance [6]. Therefore, there is an urgent need to characterize the mechanisms of breast cancer metastasis and associated biomarkers to diagnose and treat patients with breast cancer earlier.

**Fig. 1:**
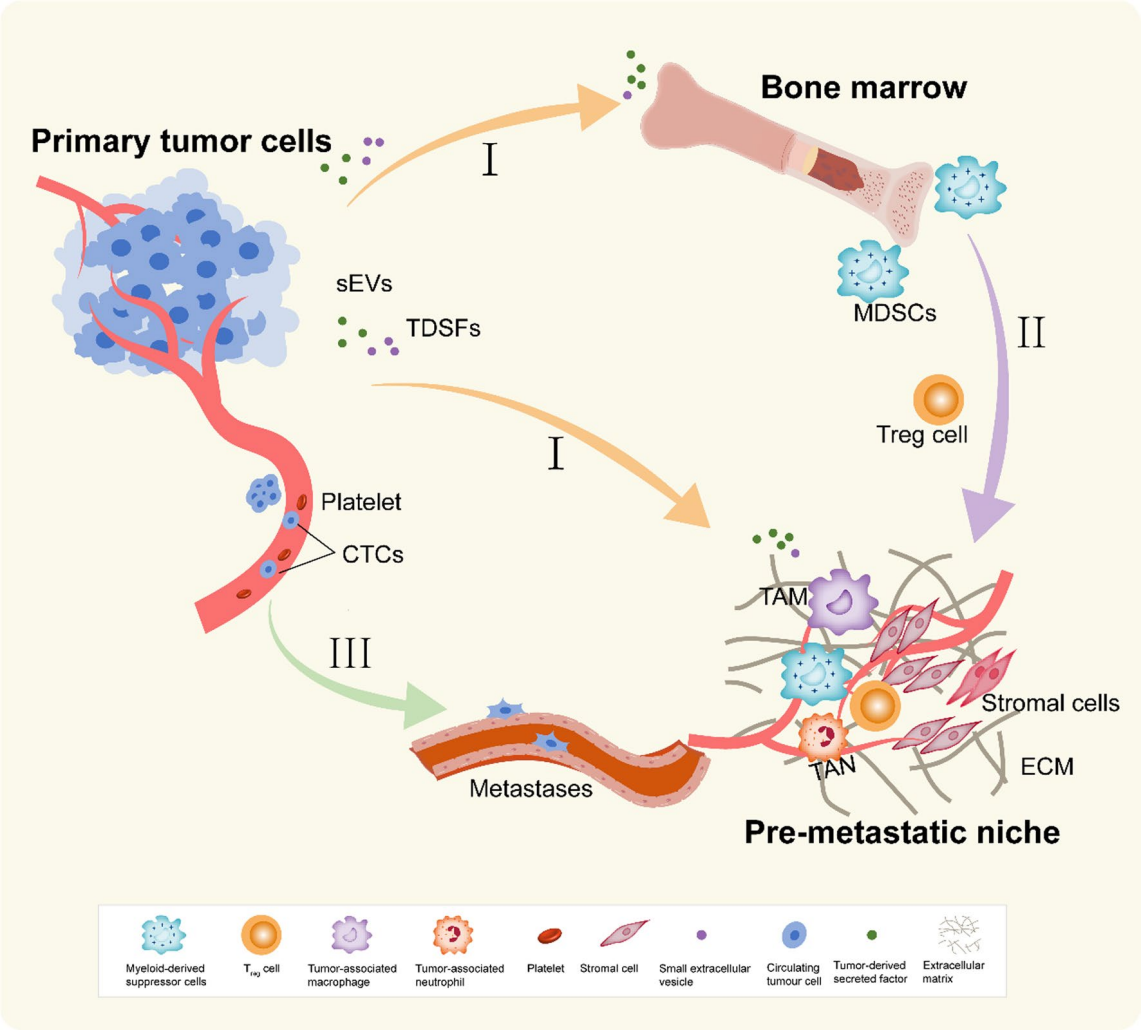
Formation of PMNs. The PMNs in cancer can be divided into three major temporal phases following a sequential order. First, the metastatic microenvironment is deternmined by the primary tumour. Second, the secondary sites recruit immunosuppressive cells. Finally, circulating tumour cells invade and colonize distant organs or tissues

Metastasis of breast cancer is characterized by heterogeneity, a feature that is largely determined by the metastatic microenvironment [7]. There are pre-metastatic niches (PMNs), which are predetermined microenvironments prior to widespread metastasis in distant organs [8, 9]. PMNs are favourable for tumour growth prior to the arrival of circulating tumour cells but are devoid of tumour cells, unlike the tumour microenvironment [9]. PMNs are initiated and established by the interaction of primary tumour-derived factors, tumour-mobilized bone marrow-derived cells and local stromal components [10]. The above three key components affect and regulate PMNs through six aspects, including immunosuppression, inflammation, angiogenesis/vascular permeability, lymphangiogenesis, organotropism, and reprogramming [11]. According to the formation of the PMN, it can be mainly divided into three stages [11] (Fig. 1). Recent findings suggest that breast cancer metastasis is likely to be mediated by the PMN [12, 13]. Breast cancer cells establish an osteogenic niche prior to osteolytic metastasis, and the growth ability of breast cancer bone metastases is regulated by pre-metastatic stromal cells [14]. Furthermore, extracellular matrix proteins, as components of the PMN, play a role in the colonization of early metastatic organs in breast cancer [15]. As important regulators of the pre-metastatic microenvironment, immune cells, including myeloid-derived suppressor cells (MDSCs), tumour-associated macrophages (TAMs), and neutrophils, provide fertile soil for breast cancer metastasis through immunosuppression, cancer cell adhesion, and angiogenesis [16, 17]. The mechanism of sEVs in PMNs has gradually come to light with in-depth research on sEVs in recent years [18, 19]. sEVs are extracellular vesicles 30–150 nm in diameter carrying nucleic acids, proteins, lipids, and metabolites, and these signatures make them diagnostic biomarkers and largely involved in tumour progression [20–22]. They act as intercellular shuttles, which are crucial both for primary tumour growth and metastatic spread [23–25]. Recent studies have confirmed that sEVs induce the establishment of PMNs in the lung and bone of breast cancer [26, 27].

In this review, we summarize the functions of sEVs in breast cancer metastasis with respect to PMN formation, aiming to identify new antimetastatic treatment targets and evaluate sEVs as predictive biomarkers of metastases.

## sEVs interact with inflammatory molecules in the PMN

Chronic inflammation affects breast cancer in a number of ways, including proliferation, survival, and migration [28]. By releasing proinflammatory cytokines, tumour cells and stromal cells can provide a secondary organ microenvironment for metastatic cells to colonize [29–31]. Thus, proinflammatory molecules are actively involved in niche formation.

Extracellular vesicles (EVs) have previously been observed to activate endothelial cells, resulting in angiogenesis that facilitates cancer metastasis [32]. Sara P Y Che reported that tissue factor (TF)-expressing EVs activate quiescent endothelial cells by activating factor X (FXa) and cleavage of protease-activated receptor 1 (PAR-1), inducing secretion of the proinflammatory factor IL-8 in breast cancer cells. Activation of endothelial cells induces a proinflammatory phenotype that promotes PMN formation and metastasis in primary tumours [33]. Another study demonstrated that tumour-derived EVs stimulated by taxanes and anthracyclines are prometastatic in breast cancer. Chemotherapy-induced tumour EVs promote proinflammatory endothelial cell activation, and chemokine (C–C motif) ligand 2 (CCL2) upregulation via a mechanism involving EV-associated annexin-A6 (ANXA6) translocation to the lung endothelium [34]. Furthermore, sEVs are also capable of increasing cytokine secretion of interleukin (IL)-6 and IL-17 by transfecting highly metastatic breast cancer cell lines with poorly metastatic ones and potentially promoting metastasis [35]. Moreover, exosomal glycoprotein 130 (gp130) is capable of being transferred to bone marrow-derived macrophages (BMDMs) via cancer cell-derived sEVs, activating the gp130-signal transducer and activator of transcription 3 (STAT3) signalling pathway to promote IL-6 production [36]. Additionally, other researchers have found that sEV-bound cytokines are key determinants of sEV-cell interactions. Upon binding to CCL-2, breast cancer cell-derived sEVs preferentially accumulate in lung tissue and are taken up by chemokine (C–C motif) receptor 2 (CCR2^+^) immune cells, contributing to the formation of PMN [37]. Consequently, sEVs and inflammatory molecules interact to form PMNs in breast cancer. Inflammatory factors are released and endothelial cells are activated by sEVs, which results in a proinflammatory response.

## sEVs drive immunosuppression or immune surveillance in the PMN

### sEV-derived PD-L1 causes immune escape

**Fig. 2:**
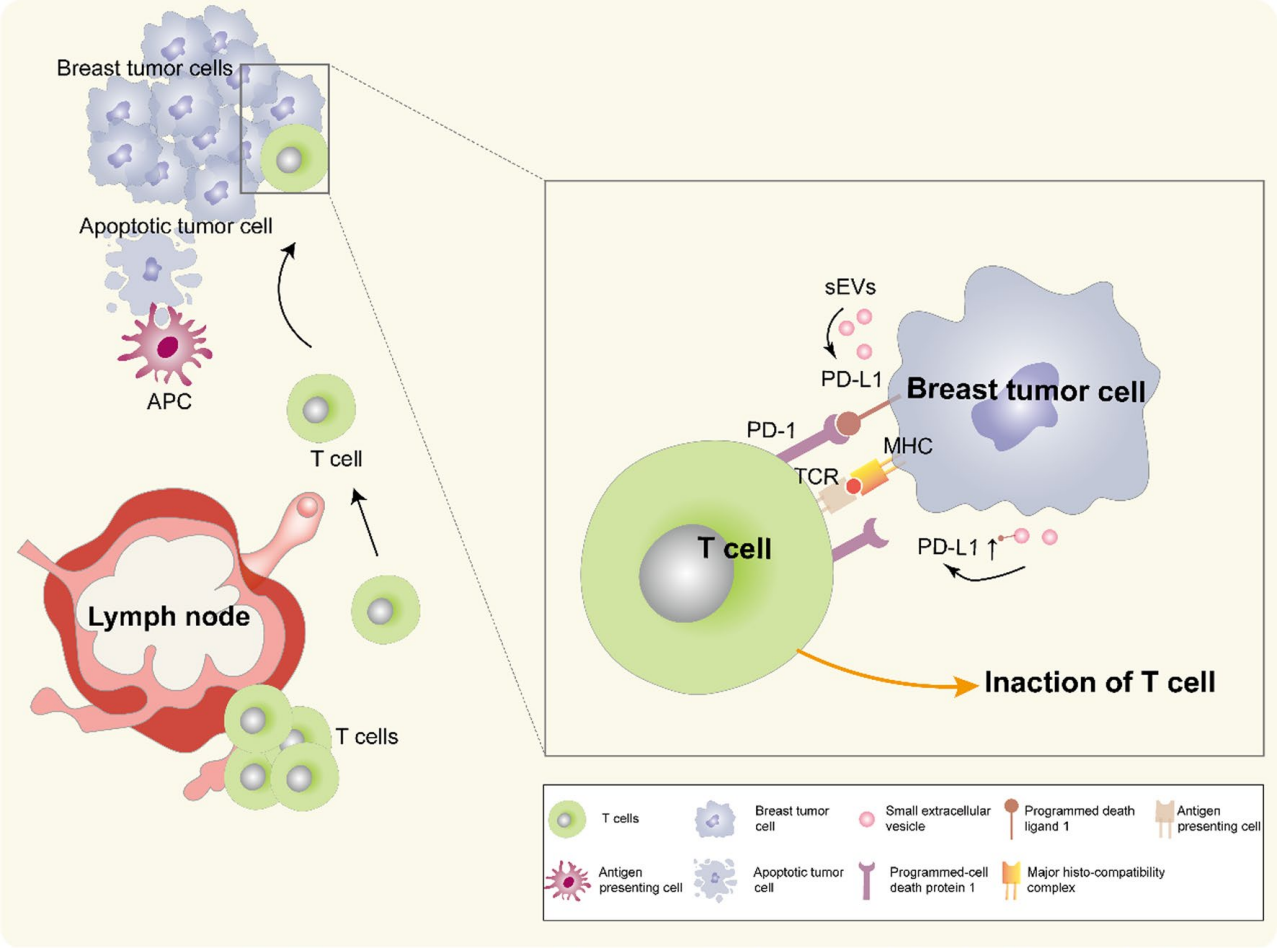
Role of exosomal PD-L1 in breast cancer. On the one hand, tumour cell surface-specific antigens are recognized by antigen-presenting cells (APCs), and apoptosis occurs; on the other hand, the combination of PD-1 on the surface of T cells and PD-L1 on the surface of tumour cells inhibits T-cell proliferation, and breast cancer cells secret sEVs that carry PD-L1 to bind to PD-1 on T cells, inhibiting T-cell activation and cell-killing activities

Programmed death ligand 1 (PD-L1) is a type I transmembrane protein that binds to its receptor, programmed-cell death protein 1 (PD-1), inactivating T cells and resulting in immune escape [38]. In recent years, PD-1, as an immune checkpoint, has attracted much attention in breast cancer treatment. Administration of anti-PD-L1 immunotherapy has become the standard treatment for breast cancer [39]. EVs from human breast cancer cells also carry immunosuppressive PD-L1, which is mostly carried by sEVs and whose level is regulated by interferon (IFN)-γ [40] (Fig. 2).

Studies suggest that in the tumour microenvironment (TME), sEVs may act as vehicles to transport PD-L1 to different cell types, thereby regulating immune surveillance [41, 42]. Morrissey demonstrated that circulating sEVs from primary breast tumours are able to be transported into the lung, increase PD-L1 expression on tissue-resident interstitial macrophages (IMs), induce an increase in PD-1^+^ T cells, and recruit MDSCs to pre-metastatic sites [43]. Therefore, exosomal PD-L1 can induce immune escape to promote tumour progression.

There has been a recent increase in studies showing that in addition to tumour cell derived sEVs, sEVs derived from other types of cells also have similar functions. A higher level of PD-L1 expression was observed after exposure to cancer-associated fibroblast (CAF)-derived sEVs in breast cancer cells, as well as miRNA-92. Apoptosis and impaired proliferation of T cells are significantly induced by increased PD-L1 expression derived from CAF-derived sEVs. Large tumour suppressor homologue 2 (LATS2) was confirmed as a target gene of miRNA-92, and in subsequent immunoprecipitation experiments, it was found that LATS2 could interact with yes-associated protein 1 (YAP1), which could bind to the enhancer region of PD-L1 after nuclear translocation, promoting transcriptional activity [44]. Researchers have demonstrated that exosomal miRNA-27A-3p is induced by endoplasmic reticulum stress to promote breast cancer immune escape by upregulating macrophage PD-L1 expression, and this effect is mediated via the MAGI2/PTEN/PI3K axis [45]. Additionally, sEVs derived from bone marrow-derived cells (BMDCs) also carry PD-L1, and effectively inhibit the response of CD8^+^ T cells [46]. In summary, the delivery of PD-L1 by sEVs could influence tumour metastasis by suppressing immune function in the pre-metastatic microenvironment, thereby contributing to PMNs.

### Inhibition of immune cell response

In pre-metastatic organs, breast cancer sEVs may suppress anticancer immune responses by inhibiting T-cell proliferation and natural killer (NK) cell cytotoxicity. In addition, sEVs derived from highly metastatic breast cancer cells are more effective at recruiting MDSCs than those from poorly metastatic cancer cells [47]. MDSCs are a group of immature myeloid cells that accumulate in cancer patients and appear in the early PMN, and immunosuppression is a key property of MDSCs [48, 49]. A study demonstrated that breast tumour-derived exosomal miRNA-200b-3p may be involved in the regulation of AKT/NF-κB/CCL2 cascades, which recruit MDSCs and lead to the construction of a metastatic microenvironment in the lung [50]. In addition, immunosuppressive cell populations can be recruited by sEVs derived from cells subjected to mechanical strain, such as macrophages [51].

### Functional injury of immune cells

sEVs can promote immune evasion of cancer cells by modulating the activity of immune cells, thus forming an immunosuppressive premetastatic microenvironment [52, 53]. 4T1 breast cancer cells secrete sEVs that block myeloid precursor cells from dividing into CD11c^+^ dendritic cells (DCs) and induce apoptosis. According to these findings, sEVs from breast cancer cells inhibit the maturation of DCs, thus facilitating immune evasion [54, 55].

### Molecular regulation

Moreover, the immune system is regulated by sEVs through their molecular functions in the PMN. MiRNA-9 and miRNA-181a found in breast cancer sEVs promote the expansion of MDSCs through their targets suppressor of cytokine signalling 3 (SOCS3) and protein inhibitor of activated STAT protein 3 (PIAS3) [56]. Breast tumour cells can regulate the production of proinflammatory cytokines by macrophages by means of sEV-mediated transfer. It has been shown that breast cancer cell-derived sEVs enhance TAM expression of IL-1β, IL-6, and TNF-αand that TANs inhibit 4T1-cell sEV secretion, resulting in a marked decrease in IL-1β, IL-6, and TNF-α.

### The heterogeneity of sEVs

Emerging evidence shows that sEVs, like tumour cells, are also heterogeneous [40, 57]. sEVs produced by highly metastatic breast cancer cells and nonmetastatic breast cancer cells are heterogeneous. On the one hand, highly metastatic breast cancer cells produce sEVs that are better at recruiting MDSCs, and on the other hand, a PMN capable of promoting metastasis is initiated in part by sEVs released by highly metastatic breast cancer cells [47].

In summary, the role of immune cells in tumourigenesis is a double-edged sword, and interestingly, the crosstalk between immune cells and cancer cells is primarily mediated by sEVs. sEVs further promote the establishment of the PMN by building an immunosuppressive microenvironment. It is not only possible for sEVs to mediate immune escape via PD-L1, but they can also cause negative effects by interfering with or damaging immune cells, and by triggering related molecules. Additionally, sEVs derived from different types of breast tumour cells show heterogeneity.

## The promoting effects of sEVs on angiogenesis and vascular permeability in the PMN

Vascular endothelial growth factor (VEGF) is a major regulator of angiogenesis, a complex process in which vessels develop from a preexisting vascular network [58]. In cancer progression, sEVs carry numerous proangiogenic biomolecules, such as VEGF, matrix metalloproteinsases (MMPs), and miRNAs, favouring metastasis to sentinel lymph nodes and distal organs [59, 60]. Sayantan found that exosomal annexinA2 (AnxA2) promotes angiogenesis and activates the p38, nuclear factor kappa-B (NF-ĸB) and STAT3 pathways to create a PMN that induces breast cancer lung and brain metastasis [61]. Proteomic analysis showed that AnxA2 was abundant in sEVs [62]. Additionally, the amount of secreted AnxA2 was positively related to the aggressiveness of breast cancer cells [63]. The level of serum exosomal AnxA2 was significantly higher in TNBCs than in ER + and HER2 + breast cancer subtypes as well as in females without breast cancer [64]. The results indicate that serum exosomal AnxA2 plays a role in angiogenesis and is linked to the aggressiveness of TNBC in aplastic anaemia (AA) women [64]. In another study, an experiment was carried out by comparing sEVs from the claudin-low TNBC cell line Hs578T and its more invasive Hs578Ts(i)8 variant. The results showed that Hs578Ts(i)_8_-derived sEVs stimulate greater vasculogenesis and angiogenesis [65]. Furthermore, MMP facilitates the assembly of new tumour blood vessels, causing the release of breast tumour cells into the circulation [66]. The aspartate β-hydroxylase (ASPH)-Notch axis regulates a range of specific sEVs to potentiate multifaceted metastasis. In breast cancer, ASPH activates Notch signalling, and Notch signalling eventually leads to sEV release, which promotes cancer spread and metastatic growth. As part of the in vitro angiogenesis procedure, tube formation was performed to determine whether sEVs participate in lymphogenesis and/or angiogenesis. MMPs are involved in maintaining breast cancer aggressiveness as downstream target genes of sEVs secreted by breast tumour cells [67].

What’s more, sEV-secreted miRNAs play a role in promoting angiogenesis. Exosomal miRNA-22-3p targeting transgelin (TAGln) promotes tumour progression and angiogenesis in vivo [68]. In addition, neutral sphyngomyeli-nase 2 (nSMase2) can activate exosomal miRNA secretion which contributes to angiogenesis in the TME [69, 70]. A study reported that when miRNA-105 is overexpressed in nonmetastatic breast tumour cells, it can induce metastasis and vascular permeability in distant organs, although miRNA-105 is also detected in the circulation at the pre-metastatic stage in early-stage breast cancer [71]. Another study confirmed that breast cancer-secreted miRNA-939 can downregulate VE-cadherin, increasing vascular permeability [72]. In MDA-MB-231 breast cancer cells, stromal interaction molecule 1 (STIM1) downregulates exosomal miRNA-145 to promote angiogenesis [73].

Before the formation of the PMN, vascular disruption is a hallmark of the initial step [9]. sEVs have been demonstrated in all these studies to promote angiogenesis and increase vascular permeability, resulting in PMN formation in breast cancer.

## sEVs are involved in stromal remodelling in the PMN

The local stromal microenvironment is one of the most important elements for the creation of a PMN in the host, and it mainly includes fibroblasts, endothelial cells, extracellular matrix (ECM) and vasculature [11]. A metastatic niche is formed through the deposition of new ECM as well as its remodeling [74, 75]. Several mechanisms are involved in sEV-mediated tumour stromal remodelling. They can promote angiogenesis by interfering with the function of endothelial barriers, and triggering the differentiation of cells in the TME into CAFs [71, 76, 77]. Exosomal miRNA-9 promotes the phenotypic transition of normal breast cancer fibroblasts to CAFs. Based on these results, transcripts involved in regulating cell motility and ECM remodelling are regulated by the exosomal vector miRNA-9 released from transfected fibroblasts [78]. CAFs play a prominent role in the invasion and metastasis of breast cancer because they account for the majority of the microenvironment [79]. Research has revealed that sEVs from breast cancer samples increase superoxide dismutase 1 (SOD1) expression in fibroblasts, which are then converted into myofibroblasts (CAF-like) [80]. Moreover, MDA-MB-231-derived sEVs promote the transformation of fibroblasts into prometastatic CAFs and increase cell contractility, one of the main hallmarks of activated CAFs in the TME promoting cancer cell invasion [81].

## sEV-mediated metastatic organotropism

In 1889, Stephen Paget proposed the famous “seed and soil” hypothesis. Cancer cells were compared to "seeds" and the site of cancer metastasis to "soil" in his hypothesis [82]. The destination of cancer metastasis is not random; that is, like "seeds", cancer cells are able to spread throughout the body but will only grow in fertile "soil" [82]. Cells from breast cancer metastasize to specific organs, known as organotropism metastasis, and are regulated by several factors, including the host–organ microenvironment, the breast cancer subtype itself and cancer cell–organ interactions [83]. However, in this process sEVs act as “fertilizers” in preparing a favourable microenvironment at future specific metastatic sites [84–86].

Hoshino found that breast cancer-derived sEVs can be used not only to predict metastatic propensity but also to identify organ sites for future metastases [30]. First, sEVs were isolated from organotropic human breast cancer cell lines, and their observations indicated that the organotropic distribution of sEVs matched the organotropy of the origin cell line. Then, the researchers discovered that organotropic tumour sEVs are potent enough to prepare premetastatic niches to facilitate metastasis. Further in-depth research showed that integrin expression patterns in sEVs determine organotropism in the lungs, liver, and brain and mediate sEV uptake into these organs. Overall, the results show that exosomal integrins can serve as a marker of organ-specific metastasis in breast cancer [30]. The presence of exosomal miRNAs in breast cancer contributes to organ-specific metastatic disease. sEV-mediated miRNA-19a promotes breast cancer brain metastasis through targeted downregulation of phosphatase and tensin homologue (PTEN) [87]. The ability of metastatic breast cancer cells to colonize the lung of poorly metastatic breast cancer cells is dependent on exosomal miRNA-200 [88]. As the above studies demonstrate, breast cancer metastasis is not random, and breast cancer-derived sEVs allow tumour cells to colonize and translocate to specific organs.

## Exosomal coding RNAs and non-coding RNAs in the PMN

In recent years, exosomal DNAs have been reported to be associated with breast cancer progression to metastases [89, 90]. Mutant DNA and mRNA are secreted by breast tumour cells via sEVs and can be integrated into heterologous cells by sEVs; for example, phosphoinositide 3-kinase alpha (PIK3CA) mutation has been demonstrated [91]. Non-coding RNAs were considered to be only intermediate molecules and not functional. In recent years, non-coding RNAs have been increasingly recognized as important regulators of cancer, including breast cancer [92, 93]. MiRNAs are small RNA molecules with a length of 18–25 nucleotides that regulate gene expression via posttranscriptional regulation, normally by inhibiting translation or by promoting the degradation of specific mRNAs [94, 95]. The various mechanisms by which exosomal miRNAs affect the PMN in breast cancer have come to light. The roles of exsomal miRNAs are listed in Table 1. Long non-coding RNAs (lncRNAs) contain more than 200 nucleotides and have attracted increasing attention [96]. Growing evidence suggests that lncRNAs have the potential to serve as diagnostic, prognostic biomarkers and therapeutic targets for breast cancer and have vital functions for the formation of PMNs [97–100]. Even so, there are no studies on the relationship between exosomal lncRNAs and the breast cancer PMN. Circular RNAs (circRNAs) have a closed ring structure and exert important biological functions as miRNA sponges [101]. There is increasing evidence linking exosomal circRNAs to TNBC. sEVs from TNBC that contain large amounts of circPSMA1 can be used to stimulate the migration and proliferation of recipient cells. Tumour-derived exosomal circPSMA1 is upregulated and favour the tumourigenesis, metastasis and immunosuppression of TNBC via the circPSMA1/miRNA-637/Akt1-β-catenin (cyclin D1) regulatory axis [102]. Another study found that circHIF1A also plays an important role in the progression and metastasis of TNBC [103]. Invadopodia of circSKA3 are involved in sEV formation, which increases tumour invadopodia and promotes breast cancer invasion [104].

**Table 1:** The role of exosomal miRNAs in breast cancer in the PMN

Donor cell	Exosomal miRNA	Function	Refs.
Breast cancer cell	miRNA-9 miRNA-181a	Upregulate in MDSCs, target SOCS3 and PIAS3	[56]
Astrocyte	miRNA-19a	Induce CCL2 upregulation, increase brain metastasis	[87]
Breast cancer cell	miRNA-105	Induce vascular permeability	[71]
Breast cancer cell	miRNA-122	Modifying glucose utilization by recipient PMN cells	[105]
Breast cancer cell	miRNA-200	Suppresses EMT, which enhances lung metastasis and colonization	[88]
nSMase2	miRNA-210	Enhanced angiogenesis	[69]
XIST_low_ breast cancer cell	miRNA-503	Trigger M1-M2 polarization of microglia (enhancing their PD-L1 expression to suppress local immunity)	[106]
Breast cancer cell	miRNA-21	Promote formation of PMN	[27]
CAF	miRNA-18b	Promote nuclear Snail ectopic activation inducing EMT	[107]
Breast cancer cell	miRNA-200b-3p	Regulate CCL2 expression in the lung	[50]
MSC	miRNA-100	Affect the vascular behaviour of endothelial cells	[108]

Collectively, the formation of PMNs in breast cancer involves both coding and non-coding RNA originating from sEVs. At present, exosomal miRNAs have attached great attention in inducing PMNs. Furthermore, the roles of exosomal lincRNAs and circRNAs in PMNs of breast cancer are gradually being revealed.

## Clinical applications mediated by sEVs

### Biomarkers for the pre-metastatic niche

Treatment of breast cancer aims to detect and stop tumour progression before metastasis or in the pre-metastatic niche. Hence, it is imperative to seek prognostic biomarkers of metastasis. Liquid biopsies are an emerging technique in the field of cancer diagnosis that analyses blood, urine, and other bodily fluids to derive a cancer diagnosis and prognosis [109]. For prognostic biomarkers of PMNs, sEVs are particularly advantageous, since they are stable, exist in body fluids, are less invasive, and are tumour-specific [110]. The use of sEVs as biomarkers could revolutionize the way breast cancer is diagnosed and treated. Additionally, they can be isolated from various body fluids, including serum, and their miRNA content reflects that of parental breast cancer cells [111]. Studies have demonstrated that miRNA-105 can be used as a prognostic blood marker for or for the early diagnosis of breast cancer metastasis [71]. Using animal models, researchers found that circulating miRNA-105 was significantly elevated at both the premetastasis and postmetastasis stages in tumour-bearing mice. Their clinical data revealed that the patient had breast cancer with distant metastasis whose concentration of miRNA-105 in their blood was also elevated significantly. This result was also confirmed in another study [112]. In addition, poor prognosis is associated with stemness- and metastasis-associated mRNAs in plasma exosomes from breast cancer patients [113]. Exosomal proteins can also serve as biomarkers for breast cancer [114, 115]. Using semiquantitative mass spectrometry to compare plasma sEVs enriched from advanced breast cancer patients with those enriched from age-matched controls, researchers found that sEV-related proteins can indicate breast cancer metastasis [116]. Some researchers have even established a breast cancer sEV database based on robust analysis of high-throughput expression data and a thorough literature review [117].

### sEVs as a novel therapeutic option

Currently, an increasing number of studies have applied sEVs as a drug delivery medium for anti-breast cancer and anti-metastasis treatment [118, 119]. With low immunogenicity, strong penetration abilities, and excellent specificity in homing the target, sEVs considerably outperform other nanoparticles in nanotherapy; therefore sEVs have been extensively used as a nanodrug carrier in the targeted drug delivery of breast cancer [120]. Different cell-derived sEVs can act as antitumour agents by transporting miRNA. Antitumour miRNAs can be targeted to breast cancer cells expressing the epidermal growth factor receptor (EGFR) by intravenous injections of sEVs [121]. Similarly, mesenchymal stem cell (MSC)-derived sEVs can deliver inhibitors of miRNA-142-3p, significantly reducing the levels of miRNA-142-3p and miRNA-150, and enhancing the transcription of target genes.

Vaccination can promote antitumour immunity, but many obstacles still stand in the way of its successful application. Moreover, applications of sEVs by researchers are making them popular in the development of anticancer vaccines. To date, most cancer vaccines based on sEVs derived from dendritic cells or tumour cells focus on the therapeutic aspects of the disease [122]. As early as 1998, researchers found that DC cell-derived sEVs activate specific cytotoxic T cells to exert an antitumour effect [123]. The above study was the first to support the use of sEVs to develop novel cell-free vaccines. Another pioneering study showed that tumour cell-derived sEVs act as a novel source of T-cell cross-priming tumour rejection antigens, activating CD8+ T cells and leading to tumour rejection in mice [124]. DC cell-derived sEVs are significant targets in tumour vaccines [125]. sEVs derived from DCs can stimulate T-cell responses directly by catalysing peptide-MHC complexes or indirectly by taking up and processing sEVs. Moreover, DC cell-derived sEVs can activate and promote the proliferation of NK cells when they interact with NKG2D ligands on the membranes of NK cells [122]. Compared to DC vaccines, DC-derived sEVs have a high level of stability and strong immunogenicity. Furthermore, DC-derived sEVs also contain more peptide-MHC I and -MHC II complexes than DCs [126]. In addition to providing a significant amount of tumour-associated antigens for antigen presentation, tumour cell-derived sEVs also carry mRNAs and non-coding RNAs that are critical for antitumour immunity [127, 128]. For instance, pioneer-translated peptides (PTPs) derived from intronic or exonic pre-mRNA act as tumour-associated antigens, which are delivered from the producing tumour cells to professional antigen presenting cells via sEVs. Thereby, PTPs further activate CD8+ T cells and inhibit tumour growth in mice [129]. Clinical trials conducted on DC-derived sEV vaccines suggest the potential for sEV-based vaccines [130]. In situ DC vaccines with tumour cell-derived sEVs as carriers activate type 1 conventional DCs (cDC1s) and cross-prime tumour-reactive CD8+ T-cell responses. A potent tumour-suppressive effect has been observed in mouse xenograft models of TNBC and patient-derived tumour organoids [131].

Furthermore, engineered sEVs have demonstrated highly potent and specific antitumour effects by activating cytotoxic T cells to destroy breast cancer cells expressing HER2 [132]. Unlike other cancers, TNBC does not express progesterone receptor (PR), ER or HER2, and delivering effective targeted therapy for TNBC remains a challenge. However, progress has been made in the application of sEV-targeted therapy for TNBC. Engineered sEVs not only enhance the antitumour effect of doxorubicin but also exhibit significant tumour targeting efficacy in TNBC [133]. sEV-based erastin preparations exert antitumour effects through ferroptosis in TNBC [134]. Additionally, sEVs have shown promise as a targeted therapy for breast cancer metastasis [135, 136].

It is thought that sEVs may become potential therapeutic targets for treating breast cancer metastasis in the future, as they function in cell-to-cell communication and influence metastatic niche formation.

## Conclusions

**Fig. 3:**
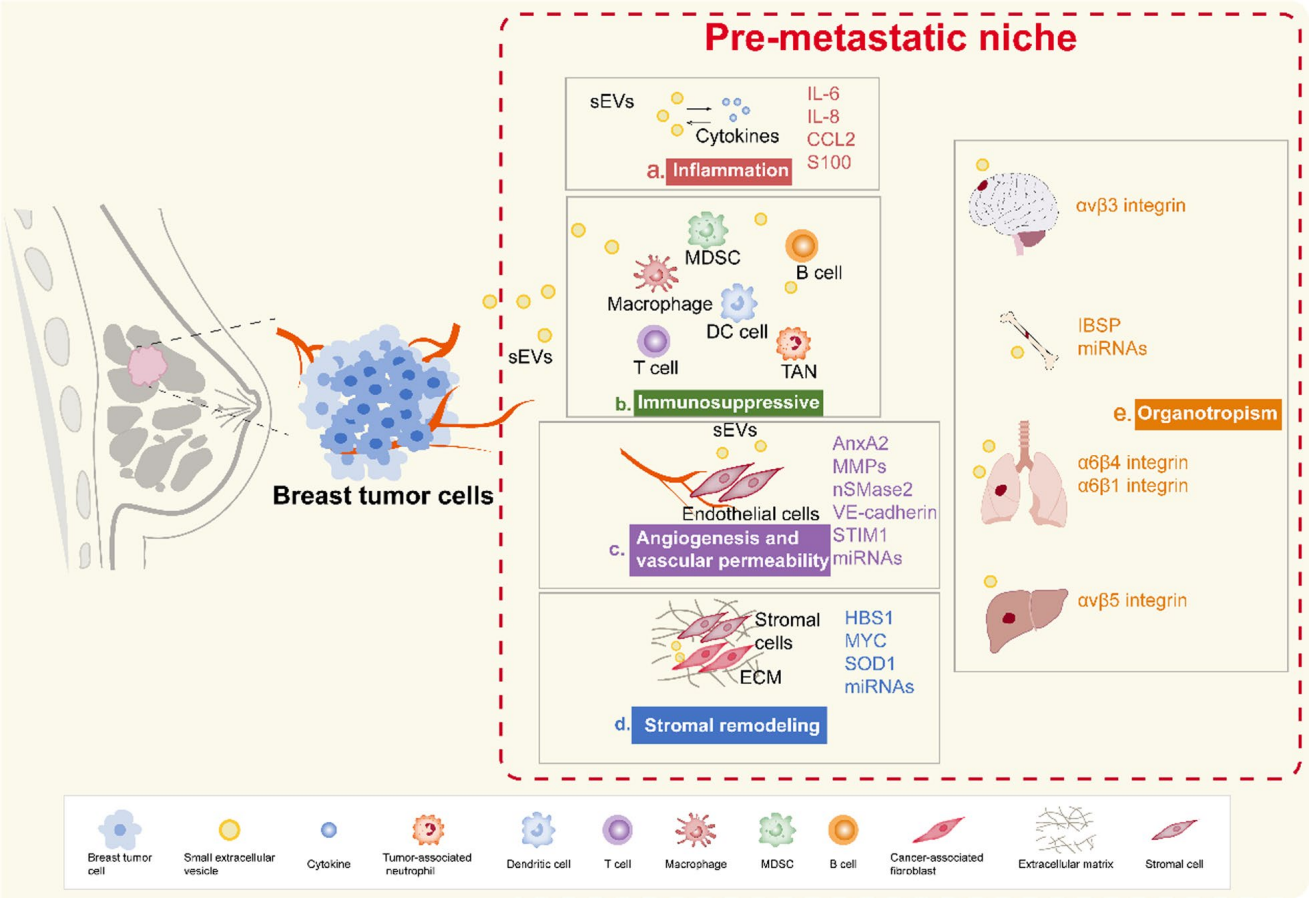
Effect of sEVs on the pre-metastatic niche in breast cancer. An overview of the effects of sEVs on the PMN of breast cancer can be summarized as follows: inflammation, immunosuppression, angiogenesis and vascular permeability, stromal remodelling and organotropism. **a** The sEVs secrete inflammatory factors, such as IL-6 and IL-8, promoting angiogenesis and recruiting immunosuppressive cells to promote the formation of breast cancer PMNs. In turn, inflammatory molecules can affect the distribution of sEVs and thus influence the PMN. **b** The sEVs not only inhibit T cells and induce immune escape by transporting PD-L1, but also exert immunosuppressive effects by recruiting MDSCs, altering DC cell activity, and transforming macrophages. sEVs also stimulate immune cells such as TANs to secrete cytokines, which suppress the antitumour immunity. **c** Through proangiogenic factors, including MMPs, and VE-cadherin, as well as miRNAs, sEVs are believed to act on angiogenesis and vascular permeability in the PMN of breast cancer. **d** In breast cancer, sEVs facilitate the turnover of CAFs to remodel the ECM and create the PMN. **e** The sEVs of breast cancer can provide a measure of organtropism, such as specific exosomal integrin combinations (there is a link between exosomal α6β4 and α6β1 integrins and lung metastasis/exosomal αvβ5 integrin with liver metastasis/exosomal αvβ3 integrin with brain metastasis). In addition, exosomal IBSP and miRNAs are involved in breast cancer brain metastasis

In summary, sEVs, by participating in cell-to-cell communication, play a momentous role in breast cancer metastasis through PMNs. sEVs can interact with inflammatory molecules to promote the formation of the PMN. In addition, sEVs can influence the establishment of the PMN from multiple aspects, including driving immunosuppression and immune surveillance, promoting angiogenesis and vascular permeability, activating stromal cells and remodelling of the ECM, and determining organotropism metastasis (Fig. 3). Non-coding RNAs in sEVs, especially miRNAs, are constantly being recognized and are closely related to the metastatic niche [137]. It may be possible to herald (or prognosticate) metastases by detecting miRNAs, thereby inhibiting the occurrence of metastases. Likely in the next, sEVs will increasingly be used in the treatment of breast cancer metastasis.

## Data Availability

Not applicable.

## Abbreviations

TNBC: Triple-negative breast cancer
PMN: Pre-metastatic niche
MDSCs: Myeloid-derived myeloid suppressor cells
TAMs: Tumour-associated macrophages
sEV: Small extracellular vesicle
TF: Tissue factor
FXa: Activating factor X
PAR-1: Protease-activated receptor 1
CCL2: Chemokine (C–C motif) ligand 2
ANXA6: Annexin-A6
Gp130: Glycoprotein 130
BMDM: Bone marrow-derived macrophage
CCR2: Chemokine (C–C motif) receptor 2
STAT3: Signal transducer and activator of transcription 3
PD-L1: Programmed death ligand 1
PD-1: Programmed-cell death protein 1
IFN: Interferon
TME: Tumour microenvironment
IMs: Interstitial macrophages
CAF: Cancer-associated fibroblast
LATS2: Large tumour suppressor homolog 2
YAP1: Yes-associated protein 1
BMDCs: Bone marrow-derived cells
NK: Natural killer cell
DC: Dendritic cell
SOCS3: Suppressor of cytokine signaling 3
PIAS3: Protein inhibitor of activated STAT protein 3
PTEN: Phosphatase and tensin homolog
PIK3CA: Phosphoinositide 3-kinase alpha
TAGln: Transgelin
NF-ĸB: Nuclear factor kappa-B
VEGF: Vascular endothelial growth factor
MMP: Matrix metalloproteinase
AnxA2: Annexina2
ER: Estrogen receptor
PR: Progesterone receptor
HER2: Human epidermal growth factor receptor 2
AA: Aplastic anemia
ASPH: Aspartate β-hydroxylase
nSMase2: Sphyngomyelinase 2
STIM1: Stromal interaction molecule 1
ECM: Extracellular matrix
SOD1: Superoxide dismutase 1
miRNA: MicroRNA
lncRNA: Long non-coding RNA
circRNA: Circular RNA
IL: Interleukin
EGFR: Epidermal growth factor receptor
MSC: Mesenchymal stem cell
cDC1s: Type 1 conventional DCs
TDSFs: Tumour-derived secreted factors
CTCs: Circulating tumour cells
Treg: Regulatory T cell
TAM: Tumour-associated macrophages
TAN: Tumour-associated neutrophils
TCR: T cell receptor
MHC: Major histo-compatibility complex
APC: Antigen presenting cell
IBSP: Integrin-binding sialoprotein
PTPs: Pioneer-translated peptides
